# Decreased Survival of Invasive Ductal Breast Cancer Patients With Two Macrometastatic Lymph Nodes Among Few Resected Ones: Should Current Sentinel-Lymph-Node Guidelines Be Revised?

**DOI:** 10.3389/fonc.2021.669890

**Published:** 2021-07-19

**Authors:** Felipe A. C. Luz, Rogério A. Araújo, Marcelo J. B. Silva

**Affiliations:** ^1^Center for Projects, Prevention and Research in Cancer at the Hospital do Câncer in Uberlândia, Uberlândia, Brazil; ^2^Laboratory of Tumors Osteoimmunology and Immunology, Institute of Biomedical Sciences, Federal University of Uberlândia, Uberlândia, Brazil; ^3^Department of Clinical Medicine, Faculty of Medicine, Federal University of Uberlândia, Uberlândia, Brazil

**Keywords:** breast neoplasm, lymph node metastasis, sentinel lymph node biopsy, neoplasm staging, nomograms

## Abstract

**Purpose:**

Sentinel-lymph-node (SLN) biopsy (SLB) is an efficient and safe axillary surgical approach with decreased morbidity than total axillary lymph node dissection (ALND) in initial patients (T1–T2). Current guidelines strongly suggest avoiding completion of ALND in patients with one or two positive SLNs that will be submitted to whole-breast radiation therapy, but must be done when three SLNs are affected.

**Methods:**

We performed a SEER-based study with breast invasive ductal carcinoma patients treated between 2010 and 2015. Optimal cutoffs of positive LNs predictive of survival were obtained with ROC curves and survival as a continuous variable. Bias was reduced through propensity score matching. Cox regression was employed to estimate prognosis. Nomograms were constructed to analyze the predictive value of clinicopathological factors for axillary burden.

**Results:**

Of 43,239 initial patients that had one to three analyzed LNs, only 425 had two positive LNs and matched analysis demonstrated no survival difference versus pN2 patients [HR: 0.960 (0.635–1.452), *p =* 0.846]. The positive-to-analyzed LN proportion demonstrated a strong prognostic factor for a low rate (1 positive to ≤1.5 analyzed) [HR = 1.567 (1.156–2.126), *p =* 0.004], and analysis derived from the results demonstrated that a “negative LN margin” improves survival. Nomograms shows that tumor size is the main factor of axillary burden.

**Conclusion:**

Macrometastasis of two LNs is a poor prognostic factor, similar to pN2, in SLNB (-like) patients; more extensive studies including preconized therapies must be done in order to corroborate or refute the resistance of this prognostic difference in patients with two macrometastatic lymph nodes within few resected.

## Introduction

In initial (T1–T2) breast cancer patients with clinically negative axilla, the sentinel lymph node (SLN) biopsy (SLNB) has proven safe and not inferior compared to axillary lymph node dissection (ALND) and should be considered the standard of care in those patients due to its reduced resultant morbidity (1).

Negativity of all SLNs, irrespective of how many are resected, has been extensively proven to have similar prognosis to the ALND approach, discarding completing ALND in those patients. Additionally, current guidelines also recommend omission of ALND completion in patients with one or two macrometastatic SLNs with planned breast-conserving surgery (BCS) followed by whole-breast irradiation (2). However, several cornerstone clinical trials were criticized for modeling and statistical power issues (3).

Based on the SEER database, this study analyzed the prognostic role of two macrometastatic lymph nodes (LNs), according to the number of resected LNs, to assess possible biasing of current management.

## Material and Methods

This retrospective observational study used data provided by the Surveillance, Epidemiology, and End Results (SEER) database [18 registries, Nov 2019 (2000–2017)] of patients treated between 2010 and 2015. The database was analyzed under the ID 10068-Nov2019 authorized to Felipe Andrés Cordero da Luz.

The staging and TNM used were derived from the seventh edition of AJCC. The molecular subtypes were derived from the qualitative expression of hormone receptor (ER and/or PR) and HER2. Ethnicity was based on the classification of White, Black and others. Age was recoded as <35, ≥35 and ≤70 and >70 years.

### Inclusion and Exclusion Criteria

Female patients with invasive ductal carcinoma (IDC) NOS/TNE (Histologic Type ICD-O-3 8500/3) breast cancer (C50.0–C50.9) were included. Patients submitted to neoadjuvant therapy were excluded.

Patients were excluded according to the following criteria: more than one primary cancer; unreported age; did not undergo surgery nor were staged only at autopsy; bilateral cancer; lysed/destroyed tumor; absence of analyzed tumor; metastatic disease; no report of LNs removed; no report of LNs analyzed; only clinically classified axilla; N1mic; no criteria for staging by AJCC; type of surgery not reported; histological grade not reported; missing ER data; missing PR data; missing HER2 data; observation time <6 months; neoadjuvant therapy; and/or unreported death.

The patients were classified as a neoadjuvant scheme by classifications 5 and 6 of the variables CS Tumor Size/Ext Eval and CS Reg Node Eval/CS Lymph Nodes Eval, and classifications 255, 257, 290, 510, 610 and 810 in CS Lymph Nodes.

The following factors were utilized to retrieve the 99,240 patients from the SEER database:

{Race, Sex, Year Dx.Sex} = ' Female'
AND {Site and Morphology.ICD-O-3 Hist/behav} = '8500/3: Infiltrating duct carcinoma, NOS'
AND {Race and Age (case data only).Race recode (White, Black, Other)} != ' Unknown'
AND {Stage - 7th edition.Derived AJCC M, 7th ed (2010-2015)} = 'M0','M0(i+)'
AND {Stage - 7th edition.Derived AJCC Stage Group, 7th ed (2010-2015)} != '0','0a','0is','IV','IVNOS','IVA','IVA1','IVA2','IVB','IVC','NA','OCCULT','UNK Stage','Blank(s)'
AND {Extent of Disease.Breast Subtype (2010+)} = 'HR+/HER2+ (Luminal B)','HR-/HER2+ (HER2 enriched)','HR+/HER2- (Luminal A)','HR-/HER2- (Triple Negative)'
AND {Site and Morphology.Grade} = 'Well differentiated; Grade I','Moderately differentiated; Grade II','Poorly differentiated; Grade III','Undifferentiated; anaplastic; Grade IV'
AND {Therapy.Reason no cancer-directed surgery} = 'Surgery performed'
AND {Site and Morphology.Primary Site - labeled} = 'C50.0-Nipple','C50.1-Central portion of breast','C50.2-Upper-inner quadrant of breast','C50.3-Lower-inner quadrant of breast','C50.4-Upper-outer quadrant of breast','C50.5-Lower-outer quadrant of breast','C50.6-Axillary tail of breast','C50.8-Overlapping lesion of breast','C50.9-Breast, NOS'
AND {Site and Morphology.Laterality} != 'Only one side - side unspecified','Bilateral, single primary','Paired site: midline tumor','Paired site, but no information concerning laterality'
AND {Multiple Primary Fields.Sequence number} = 'One primary only'
AND {Cause of Death (COD) and Follow-up.Survival months flag} = 'Complete dates are available and there are more than 0 days of survival'
AND {Cause of Death (COD) and Follow-up.SEER other cause of death classification} != 'N/A not seq 0-59'
AND {Cause of Death (COD) and Follow-up.Survival months} != 0-5
AND {Stage - 7th edition.Derived AJCC T, 7th ed (2010-2015)} != 'T0','Ta','Tis','Tispu','Tispd','T1mic','NA','TX','Blank(s)'
AND {Stage - 7th edition.Derived AJCC N, 7th ed (2010-2015)} != 'N1mi','N1NOS','NA','NX','Blank(s)'
AND {Extent of Disease.ER Status Recode Breast Cancer (1990+)} != 'Unknown'
AND {Extent of Disease.PR Status Recode Breast Cancer (1990+)} != 'Unknown'
AND {Extent of Disease.Regional nodes examined (1988+)} != 99
AND {Extent of Disease.Regional nodes positive (1988+)} != 95-99
AND {Extent of Disease.CS tumor size (2004-2015)} = 1-995
AND {Extent of Disease.CS extension (2004-2015)} != 950-999
AND {Extent of Disease.CS lymph nodes (2004-2015)} != 130,150,155,255,257,290,510,610,735,740,745,810
AND {Extent of Disease.CS Reg Node Eval (2004-2015)} != 0-2,5-6,8-9
AND {Extent of Disease.CS Tumor Size/Ext Eval (2004-2015)} != 0-2,5-6,8-9
AND {Extent of Disease.CS Mets Eval (2004-2015)} != 9
AND {Extent of Disease.CS mets at dx (2004-2015)} = 0-7
AND {Extent of Disease.CS site-specific factor 6 (2004+ varying by schema)} != 888-988
AND {Therapy.RX Summ--Surg Prim Site (1998+)} != 19,90-99
AND {Extent of Disease.CS site-specific factor 7 (2004+ varying by schema)} != 998-999
AND {Extent of Disease.CS site-specific factor 15 (2004+ varying by schema)} != 988-999

After applying the filters, 99,240 patients were eligible. Analyses were primarily performed within those patients, but the sample numbers 90,983 and 90,142 were used in analyses considering tumor location and tumor size in millimeters, respectively. The rational flow of patients included in each analysis is depicted in **Supplementary Figure 1**.

### Sentinel Lymph Node

A surrogate of SLNB was established using the number of analyzed LNs due to the lack of reports in the SEER database (4). Patients were classified as SLNB-like (SLNBL) if they had up to three LNs analyzed for three important reasons: 1) in the SLNB approach, a minimum of two SLNs is recommended to resect (1), but usually ranges from one to three LNs (5–7); 2) the involvement of three LNs is indicative of complementary ALND (2, 8); and 3) the potential residual disease could cause misclassification of the N category according to the AJCC guidelines.

### Statistical Analysis

Statistical analyses of survival, propensity score matching (PSM), generalized linear models with counting response and binary logistic regression were performed using the software IBM SPSS v25.0. The survival curves with continuous predictor and their optimal cutoff point were established using the software Jamovi v1.6.5.0. The ROC curves were generated using MedCalc v19.3.1 and Jamovi v1.6.5.0. Chi-squared test was performed using GraphPad Prism 8.0. A nomogram was built using the nomolog program (9) after logit command in the software STATA 16.0. In all analyses, statistical significance was defined as p <0.05.

The Kaplan–Meier estimator was employed to analyze the proportionality of risks as a prerequisite for considering the variable in the Cox regression model. To determine an independent prognostic factor, all significant variables were inserted into Cox’s multivariate regression model. The predictive factors for LN involvement, Poisson, and negative binomial regression with log link were analyzed according to dispersion of data; overdispersion was considered when value/df >1.2. Binary logistic regression was performed using the Stepwise Wald method with an input and output *p*-value of 0.05. For PSM, the categorical variables of race, age, T, histological grade, molecular subtype, and N and type of surgery if applicable, were used with a tolerance of 0.000001, although no continuous or discrete variable was used in the pairing. The ROC curves were generated by the DeLong method and the best cutoff point was estimated by using the Youden’s J index. The AUC was considered weak in the range of 0.5–0.7, moderate between 0.7 and 0.8, and strong above 0.8.

## Results

We analyzed 99,240 patients, of which 63,569 were initial (T1–T2) and submitted to breast-conserving surgery (BCS). This latter group of patients was analyzed with a median follow-up of 53 months (6–95). A strong association was observed between SLNBL and pN0 compared to non-SLNBL [OR = 8.088 (7.706–8.488), *p <*0.0001]. Lymph node involvement is described in **Supplementary Tables 1** and **2**. The number of analyzed LNs was not a prognostic factor [HR = 0.922 (0.850–1.000), non-SLBNL compared to SLBNL, *p =* 0.051] by multivariate Cox regression.

### Two Positive Lymph Nodes Are Predictive of Poorer Survival in SLNBL Patients

Given that the presence of pN1 patients could imply decreased survival of SLNB, we tested how many positive LNs would be predictive of decreased survival. By the ROC curve [AUC = 0.533 (0.503–0.564), *p =* 0.032], the best cutoff was >1 positive LN (J index 0.06483; sensitivity 23.90%. specificity 82.58%) in SLNBL patients. The continuous explanatory survival analysis demonstrated that the number of positive LNs is a predictor of survival [HR = 1.12 (1.03–1.230 *p =* 0.010] and the same cutoff was obtained. Therefore, we reclassified the patients according to nodal burden in each group using this cutoff and compared them to pN2. As shown in **Table 1**, the positivity of two LNs has a similar survival prognosis as compared to the pN2 category, but only in SLNBL patients.

**Table 1:** Prognostic factor of nodal burden according to lymph nodes analyzed (*n =* 63,074).

Factor	Univariate		Multivariate^1^	
	HR (95%CI)	*p*	HR (95%CI)	*p*
Reclassification				
N2	1		1	
Non-SLNBL (two or three positive LNs)	0.644 (0.530–0.783)	<0.0005	0.694 (0.571–0.844)	<0.0005
Non-SLNBL (one positive LN)	0.585 (0.483–0.709)	<0.0005	0.649 (0.536–0.787)	<0.0005
SLNBL (two positive LNs)	0.948 (0.678–1.325)	0.756	0.958 (0.685–1.339)	0.800
SLNBL (one positive LN)	0.624 (0.504–0.772)	<0.0005	0.763 (0.616–0.944)	0.013
Non-SLNBL (N0)	0.344 (0.292–0.406)	<0.0005	0.433 (0.367–0.511)	<0.0005^*^
SLNBL (N0)	0.360 (0.310–0.418)	<0.0005	0.457 (0.392–0.533)	<0.0005^*^

^1^Covariates with age, ethnicity, T, molecular subtype and grade.

^*^No significant difference (p = 0.245).

To decrease biases, we matched patients by propensity score (PSM). Four hundred and thirty SLNBL patients were matched to corresponding non-SLNBL patients, showing again an increased death risk of about 70% (**Table 2**); as expected, this difference was more pronounced in the matched analysis of patients including only 2 macrometastatic lymph nodes (427 matched patients by each arm; Log Rank p = 0.001) [multivariate HR = 2.170 (1.327 - 3.550, p = 0.002)] (data not shown). Furthermore, 428 SLNBL patients matched to corresponding pN2 patients presented a similar prognostic factor (**Table 2**). Additionally, 1,352 non-SLNBL patients with two or three positive LNs were matched to corresponding pN2 patients, showing, again, a better prognosis for the first group (**Table 2**).

**Table 2:** Prognostic factor of nodal burden according to lymph nodes analyzed after propensity score matching (PSM).

Factor	Univariate		Multivariate^1^	
	HR (95%CI)	*p*	HR (95%CI)	*p*
Reclassification				
N2 (*n* = 428)	1		1	
SLNBL (two positive LNs) (*n* = 428)	1.084 (0.716–1.642)	0.703	1.042 (0.689–1.576)	0.846
Non-SLNBL (two or three positive LNs) (*n* = 430)	1		1	
SLNBL (two positive LNs) (*n* = 430)	1.787 (1.124–2.841)	0.014	1.678 (1.058–2.660)	0.028
N2 (*n* = 1,352)	1		1	
Non-SLNBL (two or three positive LNs) (*n* = 1,352)	0.640 (508–806)	<0.0005	0.611 (0.485–0.769)	<0.0005
SLNBL (one positive LN) (*n* = 425)	1		1	
SLNBL (two positive LNs) (*n* = 425)	1.421 (0.895–2.258)	0.137	1.284 (0.807–2.041)	0.291
N2 (*n* = 1,199)	1		1	
SLNBL (one positive LN) (*n* = 1,199)	0.805 (0.623–1.038)	0.095	0.791 (0.613–1.021)	0.072

^1^Covariates with age, ethnicity, T, molecular subtype and grade.

In addition, we matched the SLNBL and non-SLBNL patients to pN2 only submitted to ALND (>10 examined LN). Four hundred and nine SLNBL matched to corresponding pN2 patients and multivariate analysis showed similar results [HR = 1.334 (0.858–2.075), *p =* 0.201 for SLNBL compared to N2] (data not shown). One thousand and five non-SLNBL were matched to corresponding pN2 patients and multivariate analysis found similar results [HR = 0.697 (0.535–0.909), *p =* 0.008 for non-SLNBL compared to N2] (data not shown).

Likewise, we matched SLNBL patients presenting only one positive LN with those with a poorer prognosis. Approximately 425 SLNBL patients with only one positive LN matched to corresponding SLNBL patients with two positive LNs revealed a similar prognosis (**Table 2**). Furthermore, the matching of 1,199 of these patients to corresponding pN2 patients found no prognostic difference (**Table 2**).

### Determining a Number of Safe Lymph Nodes

As those results could suggest contamination of non-analyzed metastatic LNs, we analyzed the impact of the positive versus analyzed LNs on the rate of their natural logarithmic conversion. Because of the nature of those calculations, only patients with at least two analyzed LNs and at least one positive LN were included (*n =* 8,558). By the ROC curve [AUC = 0.563 (0.542–0.584), *p <*0.0001], the best cutoff was >0.408 (J index 0.1209; sensitivity 41.87%. specificity 70.21%). The continuous explanatory survival analysis demonstrated that the ratio is a predictor of survival [HR = 1.00 (1.00–1.00), *p =* 0.015] while obtaining the same >0.408 cutoff.

We tested whether this rate could potentially have a prognostic significance after matching by propensity score only pN1 patients (*n =* 2,002). Using the Stepwise Forward Wald Cox regression model, this rate (categorical) demonstrated to be an independent prognostic factor [HR = 1.567 (1.156–2.126), *p =* 0.004].

By exponential conversion (*e* ^0,408^), this rate represents one positive LN for >1.50 analyzed, that is, at least two, four or five analyzed LNs when one, two or three are involved, respectively. Then, we divided the patients according to the number of analyzed LNs (up to three, two or three, four or five and more than five). After matching these patients by PSM, we performed Cox regression analysis. There was no difference in survival with these different quantities compared to more than five LNs (data not shown), but differences were observed when analysis were performed according to pN status (N− or N+). Although four or five LNs has a better prognostic factor in N− patients, it failed to prove protection in N+ patients (**Supplementary Table 3**). Differently, compared to more than five LNs, a trend of increased survival was observed for two or three LNs in N− patients and no difference was observed in N+ patients (**Supplementary Table 3**).

Then, we analyzed whether patients with two positive LNs had a different prognosis according to the number of analyzed LNs. However, only 292 patients with two affected LNs had four or five analyzed, without prognostic difference by multivariate analysis (data not shown). Considering those with up to five LNs analyzed, no survival difference was observed compared to pN2 [HR = 1.073 (0.764–1.505), *p =* 0.685]. Therefore, we included patients with four to ten analyzed LN (*n =* 727). After matching these patients (*n =* 822), the group with four to ten analyzed LNs had a better survival than patients with two or three analyzed LNs by multivariate analysis [HR = 0.580 (0.355–0.945), *p =* 0.029].

Similar results were obtained in patients with only one affected LN. When matching with N2 patients, patients with four or five analyzed LNs did not differ in survival by multivariate analysis (data not shown), but differed when considering four to ten LNs [HR = 0.711 (0.548–0.922), *p =* 0.010]. However, when considering patients with one to five analyzed LNs, the matched analysis demonstrated better survival compared to N2 patients by multivariate analysis [HR = 0.727 (0.574–0.919), *p =* 0.008] (compare with **Table 2**). Additionally, when excluding those patients with only one analyzed LN and considering only those with two or three analyzed, matched analysis demonstrated improved survival compared to N2 patients [HR = 0.684 (0.504–0.929), *p =* 0.015] (compared with **Table 2**). Those with only one analyzed had similar survival versus N2 in a matched analysis [HR = 0.887 (0.632–1.246), *p =* 0.490].

### Predictors of Axillary Burden

Because reduced survival in SLNBL patients could be a result of residual disease, we look for factors indicative of LN metastasis. For this purpose, we included the 90,983 patients with described tumor localization. Only significant explanatory variables were included in the final model, and the main predictor of axillary burden is tumor category (**Supplementary Table 4**).

**Figure 1:**
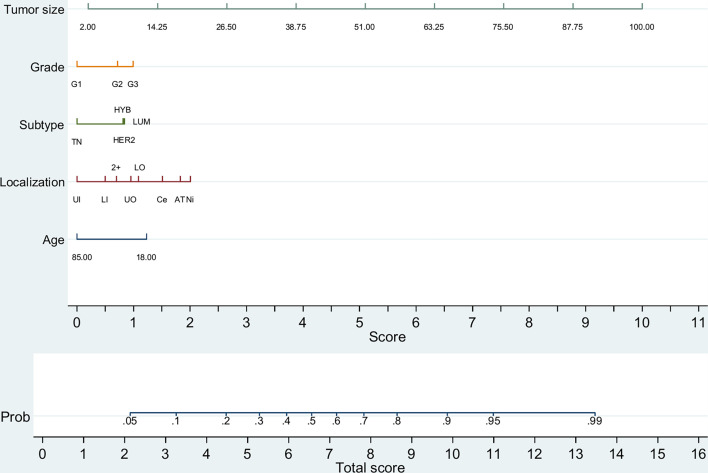
Predictive nomogram for any metastatic lymph node (pN+) (*n =* 90,142). TN, triple-negative (HR^−^/HER2^−^); LUM, luminal (HR^+^/HER2^−^); HYB, luminal hybrid (HR^+^/HER2^+^); HER2, HER2 enriched ((HR^−^/HER2^+)^); UI, upper inner quadrant; LI, lower inner quadrant; 2+, overlapping quadrants; UO, upper outer quadrant; LO, lower outer quadrant; Ce, central quadrant; Ni, nipple; AT, axillary tail. Tumor size is depicted in millimeters.

Next, we constructed two predictive nomograms for patients with tumor size up to 100 mm. Although surgery is an explanatory factor of increased analyzed LNs by binomial regression (data not shown), and consequently of positive LNs (**Supplementary Table 4**), it was excluded from the nomogram because it is not a biologically predictive factor. The first was constructed to predict any macrometastatic LN (pN+) (**Figure 1**) and the second to predict two or more macrometastatic LNs (**Supplementary Figure 2**); patients with only one analyzed LN were excluded in the second nomogram due to its biasing potential. Each nomogram provides moderate predictive accuracy [AUC = 0.765 (0.762–0.768); *p <*0.001] and [AUC = 0.778 (0.774–0.783); *p <*0.001, respectively]. As observed, tumor size is the most important predictive factor, followed by tumor location (**Supplementary Tables 5**, **6**).

**Figure 2:**
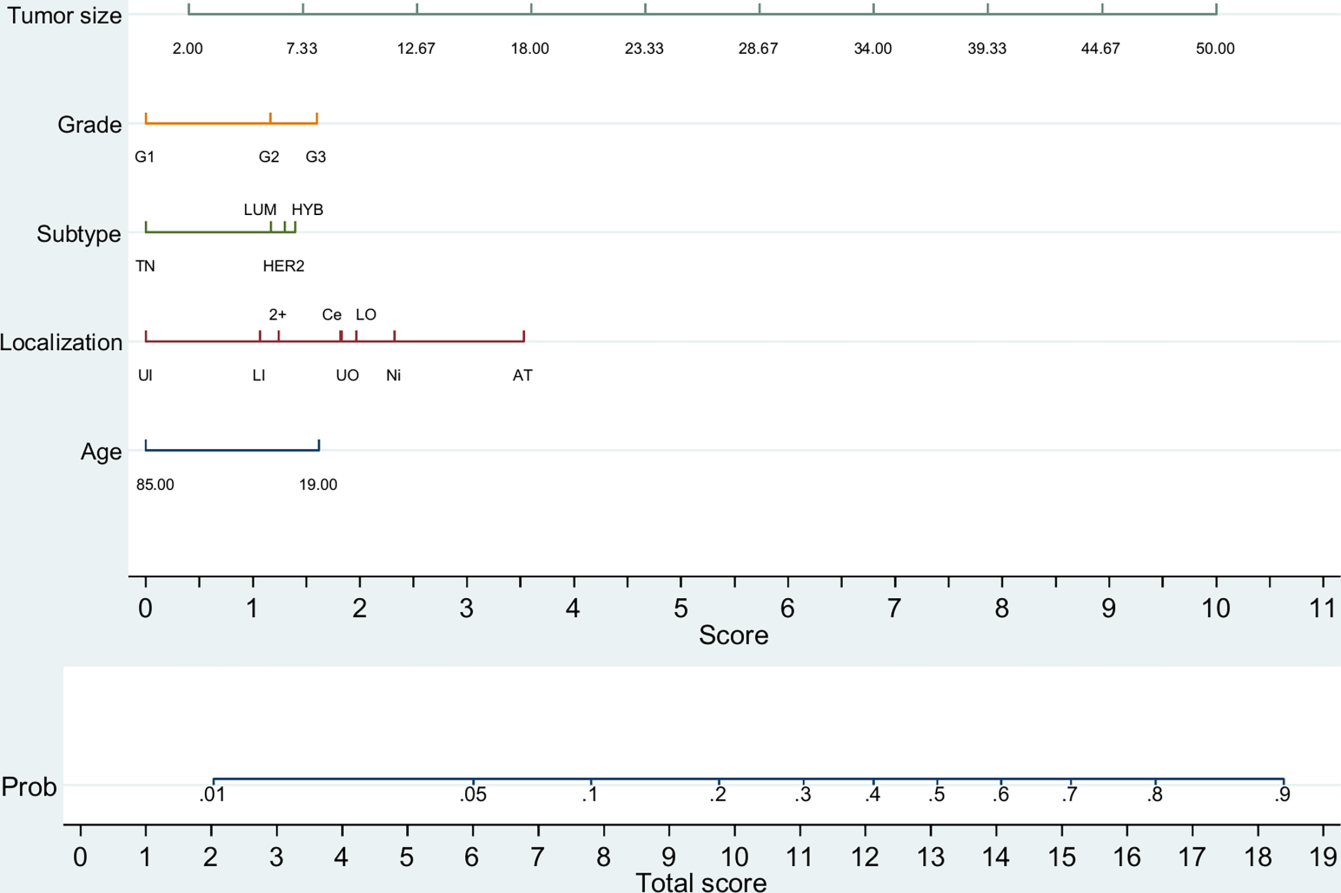
Predictive nomogram of two or more metastatic lymph nodes in initial BCS patients (*n =* 44,151). TN, triple-negative (HR^−^/HER2^−^); LUM, luminal (HR^+^/HER2^−^); HYB, luminal hybrid (HR^+^/HER2^+^); HER2, HER2 enriched ((HR^−^/HER2^+)^); UI, upper inner quadrant; LI, lower inner quadrant; 2+, overlapping quadrants; UO, upper outer quadrant; LO, lower outer quadrant; Ce, central quadrant; Ni, nipple; AT, axillary tail. Tumor size is depicted in millimeters.

For initial BCS patients, a predictive nomogram also achieved moderate accuracy [AUC = 0.764 (0.760–0.768); *p <*0.001] for predicting two or more positive LNs. Again, tumor size has the strongest predictive value, but other variables must be combined to increase the chance of the analyzed event (**Figure 2** and **Supplementary Table 7**). A nomogram for SLNBL patients, despite achieving moderate accuracy [AUC = 0.727 (0.705–0.750), *p <*0.0005], failed to predict this risk with high probability (maximum 60%; **Supplementary Figure 3**).

## Discussion

Lymph node metastasis is a major factor for survival (10). Compared to axillar ALND, the SLNB in selected patients increases quality of life without reducing overall survival (11). In line with those benefits, we observed increased overall survival in pN0 patients that had fewer resected LNs. However, several discrepancies are discussed by several authors in relation to management of patients with positive SLNs (3, 11).

The ACOSOG Z0011 and AMAROS trials are the cornerstone of reduced axillary management in early breast cancer patients. Despite some discrepancies in inclusion and exclusion criteria, both studies demonstrated the non-inferiority of the resection of these few LNs compared to ALND when few LNs present metastatic cells. The AMAROS trial showed non-inferiority of radiation therapy in patients with one or two positive SLNs compared to completion ALND, even when this procedure resulted in resection of more compromised LNs (5, 7), establishing the bases of SLNB management (2). However, in the present study, it was observed that patients with two metastatic LNs from up to three resected (SLNB-like patients) have a basal, intrinsic, decreased overall survival similar to pN2 patients in opposing data of several clinical trials.

Interestingly, during the revision of this manuscript, the 17th St. Gallen Consensus occurred, in which a clinical practice similar to current guidelines was observed only for micrometastatic SLNs, but the panelist showed uncertainty as to whether radiation therapy can replace surgery in SLN patients with one positive LN out of three (52%), and especially when two are involved (38%), demonstrating a clinical practice different from the current guidelines (12). In addition, a large retrospective analysis of a multicentric prospective study was published showing decreased overall and disease-free survivals in pN1 patients submitted to SLNB with one macrometastasis, compared to pN0 and pN1mic, only after adjusting for clinicopathological variables and adjuvant treatments, including radiation therapy. Furthermore, pN1 patients with >1 macrometastases also had decreased distant metastasis-free survival (13). The findings of the present study is in line with clinical practice adopted by the majority of oncologists worldwide.

Different prognostic factors between the ACOSOG Z0011 and AMAROS trials and increased number of micrometastatatic LNs or isolated tumor cells in the SLNB arm in the AMAROS trial can partially explain the discrepancies with our results (3). For example, it is known that triple-negative and HER2-enriched tumors possess increased radiation resistance (14–16), while the low number of triple-negative and HER2 patients in these trials could account for a portion of these results (5), as well as the insignificant role of micrometastasis in non-sentinel node burden (17). In addition, we included only patients with invasive ductal carcinoma, in contrast to major clinical trials (2). Although invasive lobular carcinoma has an increased metastatic potential toward LNs (18), they have a better survival than IDC, even in stage-matched comparisons (19–21). Thus, these different molecular biologies may have produced different results.

Several factors are predictive of non-sentinel LN metastasis, such as tumor size larger than 2 cm (22–24), number of LNs with macrometastasis (18, 24) and extracapsular node invasion (23–27), which is a warning sign that should be considered for ALND, even when only one SLN is affected (2, 8). But the SEER database lacks this report. Therefore, patients with more macrometastatic LNs have more chances of increased axillary burden that can explain why patients with two compromised LNs in SLNB surrogate group showed survival similar to pN2 patients. In fact, the presence of one LN with macrometastasis is a predictor of non-sentinel LN metastasis (23). Accordingly, the proportion between affected and removed LNs was demonstrated as a stronger factor of non-sentinel LN than the previous ones (25). We observed that this proportion is a strong prognostic factor similar to the literature (28–30), especially in pN1 patients, in the opposite direction with the favorable observations to the de-escalation of axillectomy.Results from NSABP B-32 trial, the only with sufficient power in relation to the prognostic impact of SLNB (11), help to contextualize the present problem. With balanced systemic and radiation therapy between arms the two arms (SLNB versus SLNB + ALND) (31), did not observe difference on overall survival or disease-free between arms (31, 32), but showed significantly increased risk of disease progression and a trend (p = 0.08) of increased death in patients with occult disease (32).

Even though tumor size is the most important predictive factor of increased axillary burden (≥2 LN), as observed for pT3 and pT4 tumors, as in the literature (2), the obtained nomogram for pT1–T2 patients submitted to conserving surgery is not satisfactory, probably due to the fact that the false negative rate in eligible patients for SLNB is independent of tumor size (33), the strongest factor in our models. The combination of other clinicopathological factors, such as lymphovascular invasion, extracapsular extension and clinical staging (34, 35), that are not available in the SEER database, can help to pinpoint pT1–T2 patients with positive SLNB at increased risk of higher axillary burden and also can potentially identify a subgroup of patients with larger tumors (>5 cm) who can benefit from SLNB.

Additionally, we observed that a minimum of two resected LNs implies in better staging, corroborating practical recommendations (1). In line with this, we observed a non-significant better survival rate in pN1 patients with only one dissected LN compared to pN2 patients, but significant in patients with more resected LNs. These two findings suggest that a “residual” axillary disease could have an important role in survival. Nevertheless, it is more reasonable to conclude that resecting a more significant number of lymph nodes allows a better staging, leading to less appropriate therapeutic choices in the downstaged ones (36, 37).

Our findings suggest that resection of four or five LNs may further prevent the risk of downstaging compared to the resection of two or three LNs. Yi et al. (38) observed that more than 99% of affected LNs are identified in the first five SLNs. Despite this, it is questionable whether the possible benefits could outweigh the harms, since few patients classified as SLNBL (6.3%) had one or more positive LNs. Interestingly, a recent new approach of regional lymph nodes in breast cancer therapy, that is, the partial axillary LN dissection (PALND) inferior to the intercostobrachial nerves, has demonstrated similar outcomes to ALND with decreased morbidities similar to SLNB (39). However, the nomograms failed to point out with a high degree of probability which patients may have an increased axillary burden (≥2 macrometastatic lymph nodes), which, in principle, would preclude any attempt to direct to a more specific surgical treatment of the regional lymph nodes to avoid downstaging. This study has some limitations. The SEER database has an overall sensitivity of 80% for radiation therapy data and of 68% for chemotherapy data (40). Including these variables in the analysis could potentially generate a study bias (41). Although patients with a clear mention of neoadjuvant therapy in the SEER database were excluded from analysis (4), we cannot guarantee that all patients meet the SLNB inclusion criteria as recommended by the current guidelines (2, 8), although the criteria of the principal trials also differ from current guidelines (3).

Several factors suggest that patients from this database generally received correct management according to the standard of care (42, 43), such as strong association of SLNBL with pN0, increased number of dissected LNs in those that received mastectomy (1, 2), increased survival of luminal hybrid (HR^+^/HER2^+^) patients (not shown) as a function of the benefits of anti-HER2 therapy (44–46), and decreased survival of patients submitted to mastectomy/radical surgery (data not shown) (47). However, as already mentioned, no report of SLNB or clinical staging of the axilla is available in the SEER database (4). It is important to state that, although reduced for pT1–T2 patients, the clinical axillary staging is predictive of increased axillary burden (42), but the clinical staging is inferior and does not replace the pathological staging (43). More importantly, similar results in the matched analysis by propensity score between subsets of pN1 patients and pN2 patients, and those obtained of the prognostic value of affected to removed lymph node ratio in pN1, strengthen our conclusions that an intrinsic (basal) prognostic difference exists according to the number of resected lymph nodes by a probable downstaging. Additionally, the similar prognosis of only one LN affected by cancer compared to pN2 patients corroborates these conclusions. Nevertheless, a meta-analysis or prospective study with similar criteria and a larger number of patients with two positives, macrometastatic, LNs, including the administration of systemic and radiation therapies, could corroborate the current guidelines or the present results, as well as the benefit of resection of a moderately great number of LNs.

## Conclusion

The lymph node ratio (affected to removed/analyzed) is a prognosis factor of overall survival in pN1 patients with invasive ductal carcinoma of the breast. An intrinsic survival difference was observed in patients with two macrometastatic lymph nodes according to the number of resected lymph nodes (up to three vs more than three), with a similar prognosis to pN2 disease when few (up to three) are resected, implying in downstaging. Caution must be taken, and a better-designed study must be carried out to corroborate or refute whether this baseline difference is, in fact, persistent when corrected by the recommended treatments for patients with pN1 disease undergoing SLNB.

## Data Availability Statement

The data analyzed in this study is subject to the following licenses/restrictions: SEER database is only available to those who sign the adhesion terms, available on the SEER website. Requests to access these datasets should be directed to https://seer.cancer.gov/data/access.html.

## Author Contributions

Conception and design, provision of study material or patients, collection and assembly of data, data analysis and interpretation, and manuscript writing: FL. Data revision and analysis revision: RA and MS. All authors contributed to the article and approved the submitted version.

## Funding

We thank CAPES (Coordenação de Aperfeiçoamento de Pessoal de Nível Superior) and Fundação de Amparo à Pesquisa do Estado de Minas Gerais (FAPEMIG) for supporting this work.

## Conflict of Interest

The authors declare that the research was conducted in the absence of any commercial or financial relationships that could be construed as a potential conflict of interest.
