# Corrigendum: Decreased Survival of Invasive Ductal Breast Cancer Patients With Two Macrometastatic Lymph Nodes Among Few Resected Ones: Should Current Sentinel-Lymph-Node Guidelines Be Revised?

**DOI:** 10.3389/fonc.2021.755438

**Published:** 2021-09-06

**Authors:** Felipe A. C. Luz, Rogério A. Araújo, Marcelo J. B. Silva

**Affiliations:** ^1^Center for Projects, Prevention and Research in Cancer at the Hospital do Câncer in Uberlândia, Uberlândia, Brazil; ^2^Laboratory of Tumors Osteoimmunology and Immunology, Institute of Biomedical Sciences, Federal University of Uberlândia, Uberlândia, Brazil; ^3^Department of Clinical Medicine, Faculty of Medicine, Federal University of Uberlândia, Uberlândia, Brazil

**Keywords:** breast neoplasm, lymph node metastasis, sentinel lymph node biopsy, neoplasm staging, nomograms

In the original article, there was an error. The patient selection criteria from the SEER database were incompletely entered, returning an incorrect number of patients, according to the criteria described in the study.

A correction has been made to ***Material and Methods, Inclusion and Exclusion Criteria, paragraph 5*:**

{Race, Sex, Year Dx.Sex} = ‘ Female’

AND {Site and Morphology.ICD-O-3 Hist/behav} = ‘8500/3: Infiltrating duct carcinoma, NOS’

AND {Race and Age (case data only).Race recode (White, Black, Other)}!= ‘ Unknown’

AND {Stage - 7th edition.Derived AJCC M, 7th ed (2010-2015)} = ‘M0’,’M0(i+)’

AND {Stage - 7th edition.Derived AJCC Stage Group, 7th ed (2010-2015)}!= ‘0’,’0a’,’0is’,’IV’,’IVNOS’,’IVA’,’IVA1’,’IVA2’,’IVB’,’IVC’,’NA’,’OCCULT’,’UNK Stage’,’Blank(s)’

AND {Extent of Disease.Breast Subtype (2010+)} = ‘HR+/HER2+ (Luminal B)’,’HR-/HER2+ (HER2 enriched)’,’HR+/HER2- (Luminal A)’,’HR-/HER2- (Triple Negative)’

AND {Site and Morphology.Grade} = ‘Well differentiated; Grade I’,’Moderately differentiated; Grade II’,’Poorly differentiated; Grade III’,’Undifferentiated; anaplastic; Grade IV’

AND {Therapy.Reason no cancer-directed surgery} = ‘Surgery performed’

AND {Site and Morphology.Primary Site - labeled} = ‘C50.0-Nipple’,’C50.1-Central portion of breast’,’C50.2-Upper-inner quadrant of breast’,’C50.3-Lower-inner quadrant of breast’,’C50.4-Upper-outer quadrant of breast’,’C50.5-Lower-outer quadrant of breast’,’C50.6-Axillary tail of breast’,’C50.8-Overlapping lesion of breast’,’C50.9-Breast, NOS’

AND {Site and Morphology.Laterality}!= ‘Only one side - side unspecified’,’Bilateral, single primary’,’Paired site: midline tumor’,’Paired site, but no information concerning laterality’

AND {Multiple Primary Fields.Sequence number} = ‘One primary only’

AND {Cause of Death (COD) and Follow-up.Survival months flag} = ‘Complete dates are available and there are more than 0 days of survival’

AND {Cause of Death (COD) and Follow-up.SEER other cause of death classification}!= ‘N/A not seq 0-59’

AND {Cause of Death (COD) and Follow-up.Survival months}!= 0-5

AND {Stage - 7th edition.Derived AJCC T, 7th ed (2010-2015)}!= ‘T0’,’Ta’,’Tis’,’Tispu’,’Tispd’,’T1mic’,’NA’,’TX’,’Blank(s)’

AND {Stage - 7th edition.Derived AJCC N, 7th ed (2010-2015)}!= ‘N1mi’,’N1NOS’,’NA’,’NX’,’Blank(s)’

AND {Extent of Disease.ER Status Recode Breast Cancer (1990+)}!= ‘Unknown’

AND {Extent of Disease.PR Status Recode Breast Cancer (1990+)}!= ‘Unknown’

AND {Extent of Disease.Regional nodes examined (1988+)}!= 99

AND {Extent of Disease.Regional nodes positive (1988+)}!= 95-99

AND {Extent of Disease.CS tumor size (2004-2015)} = 1-995

AND {Extent of Disease.CS extension (2004-2015)}!= 950-999

AND {Extent of Disease.CS lymph nodes (2004-2015)}!= 130,150,155,255,257,290,510,610,735,740,745,810

AND {Extent of Disease.CS Reg Node Eval (2004-2015)}!= 0-2,5-6,8-9

AND {Extent of Disease.CS Tumor Size/Ext Eval (2004-2015)}!= 0-2,5-6,8-9

AND {Extent of Disease.CS Mets Eval (2004-2015)}!= 9

AND {Extent of Disease.CS mets at dx (2004-2015)} = 0-7

AND {Extent of Disease.CS site-specific factor 6 (2004+ varying by schema)}!= 888-988

AND {Therapy.RX Summ–Surg Prim Site (1998+)}!= 19,90-99

AND {Extent of Disease.CS site-specific factor 7 (2004+ varying by schema)}!= 998-999

AND {Extent of Disease.CS site-specific factor 15 (2004+ varying by schema)}!= 988-999

The authors apologize for this error and state that this does not change the scientific conclusions of the article in any way.

## Publisher’s Note

All claims expressed in this article are solely those of the authors and do not necessarily represent those of their affiliated organizations, or those of the publisher, the editors and the reviewers. Any product that may be evaluated in this article, or claim that may be made by its manufacturer, is not guaranteed or endorsed by the publisher.

