# Supplementary material for: Decreased Survival of Invasive Ductal Breast Cancer Patients With Two Macrometastatic Lymph Nodes Among Few Resected Ones: Should Current Sentinel-Lymph-Node Guidelines Be Revised?
Source: Front Oncol. 2021 Jul 19;11:669890. doi: 10.3389/fonc.2021.669890 (PMC8327777; doi:10.3389/fonc.2021.669890)
Supplement: Supplementary file 1 [file DataSheet_1.docx]

Supplementary Material

## Supplementary Figures


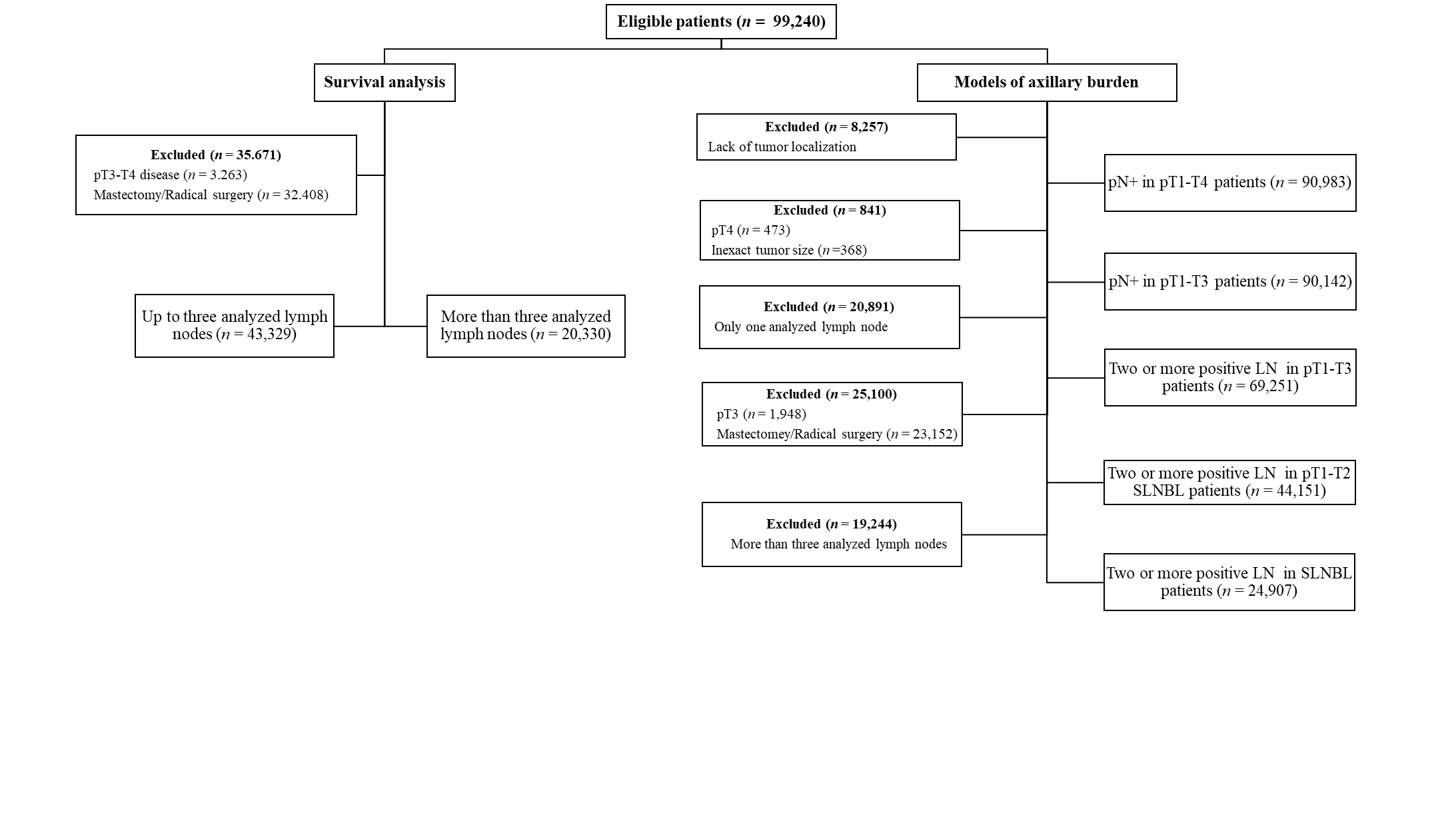


**Supplementary Figure 1.** Flowchart of patients included and excluded in each statistical analysis. Patients included in survival analysis consisted of pT1-T2 submitted to breast-conserving surgery (*n*= 63,569). Patients included in predictive models of axillary burden ranged from 90,983 to 44,151, according to different purposes.


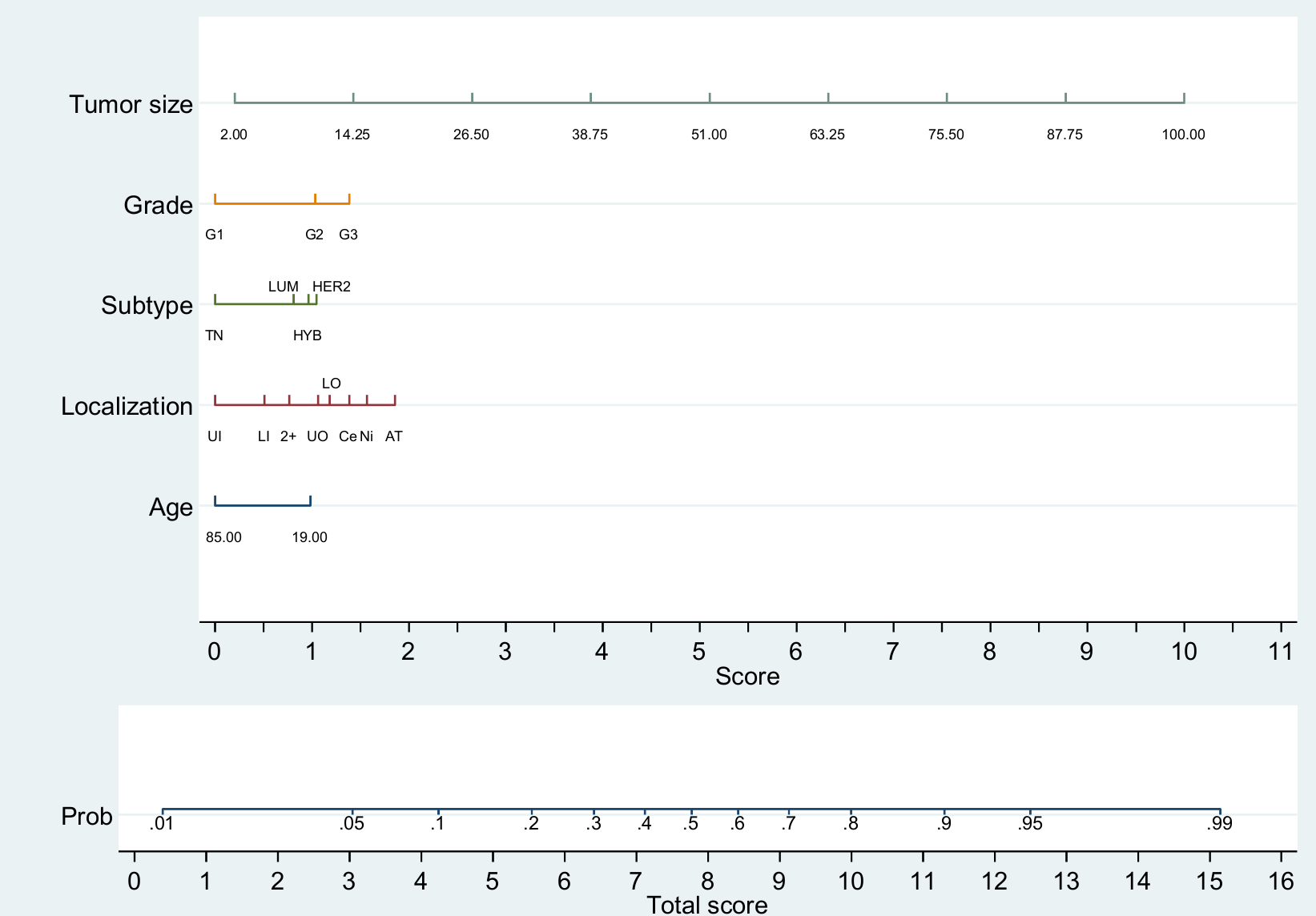


**Supplementary Figure 2**. Predictive nomogram of two or more metastatic lymph nodes in T1-T3 patients (*n*=69,251). Legend: TN - triple-negative (HR^-^/HER2^-^); LUM - luminal (HR^+^/HER2^-^); HYB - luminal hybrid (HR^+^/HER2^+^); HER2 – HER2 enriched ((HR^-^/HER2^+)^); UI – upper inner quadrant; LI – lower inner quadrant; 2+ - overlapping quadrants; UO – upper outer quadrant; LO – lower outer quadrant; Ce – central quadrant; Ni – nipple; AT – axillary tail. Tumor size is depicted in millimeters.


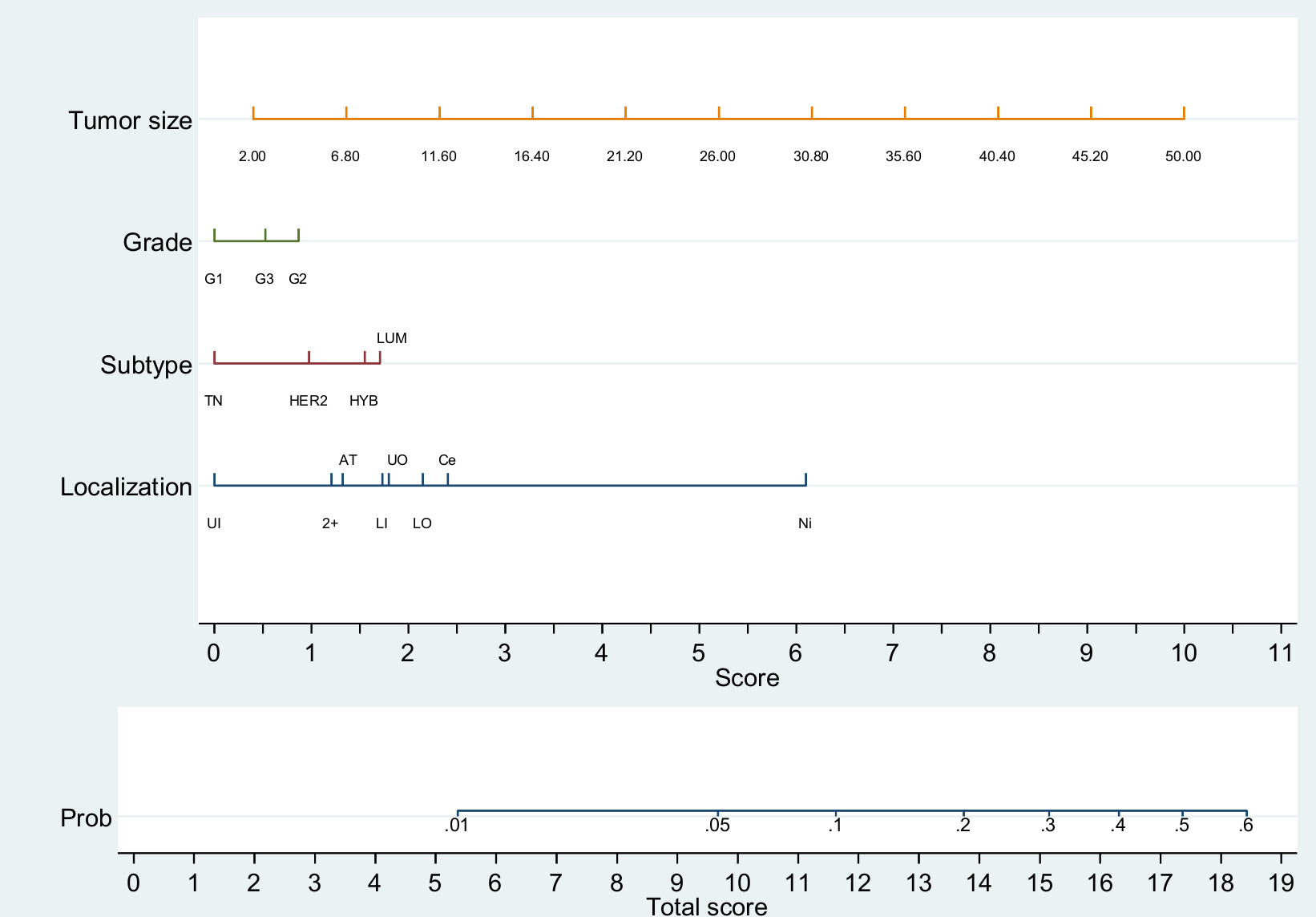


**Supplementary Figure 3**. Predictive nomogram of two or more metastatic lymph nodes in SLNBL patients (*n*=24,907). Legend: TN - triple-negative (HR^-^/HER2^-^); LUM - luminal (HR^+^/HER2^-^); HYB - luminal hybrid (HR^+^/HER2^+^); HER2 – HER2 enriched ((HR^-^/HER2^+)^); UI – upper inner quadrant; LI – lower inner quadrant; 2+ - overlapping quadrants; UO – upper outer quadrant; LO – lower outer quadrant; Ce – central quadrant; Ni – nipple; AT – axillary tail. Tumor size is depicted in millimeters.

## Supplementary Tables

**Supplementary Table 1.** pN according to the number of analyzed lymph nodes groups (*n*=63,569)

| **Group/pN** | **N0 (%)** | **N1 (%)** | **N2 (%)** | **N3 (%)** |
| --- | --- | --- | --- | --- |
| SLNBL (43,239) | 40,527 (93.7) | 2,708 (6.3) | 2 | 2 |
| Non-SLNBL (20,330) | 13,191 (64.9) | 5,323 (26.2) | 1,397 (6.9) | 419 (2.1) |

**Supplementary Table 2.** Number of positive lymph nodes in pN1 by each group

| **Group/positive lymph node (pN1)** | **1 (%)** | **2 (%)** | **3 (%)** |
| --- | --- | --- | --- |
| SLNBL (2,708) | 2,223 (82.09) | 435 (16.06) | 50 (1.85) |
| Non-SLNBL (5,323) | 2.850 (53.54) | 1.588 (24.20) | 861 (16.17) |

**Supplementary Table 3.** Prognosis factor of different lymph node number after PSM

|  | **Univariate** | | **Multivariate^1^** | |
| --- | --- | --- | --- | --- |
| **Factor** | **HR (95%CI)** | ***p*** | **HR (95%CI)** | ***p*** |
| **N-** |  |  |  |  |
| More than five | 1 |  | 1 |  |
| Up to three | 0.847 (0.714 – 1.006) | 0.058 | 0.849 (0.715 – 1.008) | 0.062 |
| More than five | 1 |  | 1 |  |
| Two or three | 0.950 (0.819 – 1.101) | 0.494 | 0.868 (0.748 – 1.007) | 0.062 |
| More than five | 1 |  | 1 |  |
| Four or five | 0.798 (0.669 – 0.953) | 0.012 | 0.802 (0.672 – 0.957) | 0.014 |
| **N+** |  |  |  |  |
| More than five | 1 |  | 1 |  |
| Up to three | 1.279 (1.045 – 1.567) | 0.017 | 1.293 (1.056 – 1.583) | 0.013 |
| More than five | 1 |  | 1 |  |
| Two or three | 1.031 (0.809 – 1.314) | 0.805 | 1.206 (0.938 – 1.551) | 0.144 |
| More than five | 1 |  | 1 |  |
| Four or five | 1.486 (1.115 – 1.979) | 0.007 | 1.524 (1.143 – 2.032) | 0.004 |

^1^Covariated with age, ethnicity, T, N, molecular subtype and grade.

**Supplementary Table 4.** Predictors of axillary burden by binomial negative regression (*n*=90,983)

| **Factor** | **OR (95% CI)^1^** | ***p*** |
| --- | --- | --- |
| **Age** | 0.999 (0.998 – 1.000) | 0.009 |
| **Ethnicity** |  |  |
| White | 1 |  |
| Black | 0.981 (0.939 – 1.024) | 0.375 |
| Others | *0.881 (0.844 – 0.919)* | *<0.0005* |
| **Surgery** |  |  |
| BCS | 1 |  |
| Mastectomy | *1.363 (1.325 – 1.402)* | *<0.0005* |
| **T** |  |  |
| T1 | 1 |  |
| T2 | *2.321 (2.254 – 2.389)* | *<0.0005* |
| T3 | *3.758 (3.533 – 3.997)* | *<0.0005* |
| T4 | *5.184 (4.586 – 5.860)* | *<0.0005* |
| **Grade** |  |  |
| G1 | 1 |  |
| G2 | *1.511 (1.449 – 1.576)* | *<0.0005* |
| G3 | *1.794 (1.714 – 1.877)* | *<0.0005* |
| **Subtype** |  |  |
| HR^+^/HER2^+^ | 1 |  |
| HR^+^/HER2^-^ | 0.991 (0.950 – 1.034) | 0.692 |
| HR^-^/HER2^+^ | *1.080 (1.008 – 1.156)* | *0.029* |
| HR^-^/HER2^-^ | *0.742 (0.702 – 0.783)* | *<0.0005* |
| **Localization** |  |  |
| UO | 1 |  |
| LO | 1.018 (0.970 – 1.068) | 0.478 |
| LI | *0.817 (0.770 – 0.868)* | *<0.0005* |
| UI | *0.632 (0.604 – 0.661)* | *<0.0005* |
| Overlapping | *0.899 (0.869 – 0.929)* | *<0.0005* |
| Central | *1.071 (1.009 – 1.138)* | *0.024* |
| Nipple | 1.244 (0.998 – 1.552) | 0.052 |
| Axillary tail | *1.331 (1.130 – 1.596)* | *0.001* |

^1^The natural logarithm of analyzed LNs was used as an offset value

Legend: BCS – breast conserving surgery; UO – upper outer quadrant; LO – lower outer quadrant; LI – lower inner quadrant; UI – upper inner quadrant.

**Supplementary Table 5.** Predictors of any metastatic lymph node by logistic regression (*n*=90,142)

| **Factor** | **Coef. (95% CI)** | **Z** | ***p*** |
| --- | --- | --- | --- |
| **Constant** | -2.151 (-2.262 – -2.039) | -37.89 | <0.0005 |
| **Tumor size** | 0.066 (0.065 – 0.068) | 83.58 | <0.0005 |
| **Localization** |  |  |  |
| UO | 0 |  |  |
| LO | 0.091 (0.029 – 0.152) | 2.91 | 0.004 |
| LI | -0.300 (-0.378 – -0.223) | -7.67 | <0.0005 |
| UI | -0.632 (-0.690 – -0.574) | -21.47 | <0.0005 |
| Overlapping | -0.167 (-0.211 – -0.124) | -7.52 | <0.0005 |
| Central | 0.374 (0.295 – 0.453) | 9.26 | <0.0005 |
| Nipple | 0.702 (0.413 – 0.991) | 4.76 | <0.0005 |
| Axillary tail | 0.583 (0.376 – 0.789) | 5.53 | <0.0005 |
| **Age** | -0.012 (-0.013 – -0.011) | -17.27 | <0.0005 |
| **Grade** |  |  |  |
| G1 | 0 |  |  |
| G2 | 0.478 (0.426 – 0.529) | 18.18 | <0.0005 |
| G3 | 0.661 (0.604 – 0.719) | 22.65 | <0.0005 |
| **Subtype** |  |  |  |
| HR^+^/HER2^+^ | 0 |  |  |
| HR^+^/HER2^-^ | 0.008 (-0.048 – 0.065) | 0.33 | 0.741 |
| HR^-^/HER2^+^ | 0.016 (-0.079 – 0.112) | 0.29 | 0.770 |
| HR^-^/HER2^-^ | -0.555 (-0.629 – 0.481) | -14.40 | <0.0005 |

Legend: UO – upper outer quadrant; LO – lower outer quadrant; LI – lower inner quadrant; UI – upper inner quadrant.

**Supplementary Table 6.** Predictors of two or more positive lymph node by logistic regression in T1-T3 patients (*n*=69,251)

| **Factor** | **Coef. (95% CI)** | **Z** | ***p*** |
| --- | --- | --- | --- |
| **Constant** | -2.793 (-2.934 – -2.651) | -38.70 | <0.0005 |
| **Tumor size** | 0.062 (0.060 – 0.064) | 71.02 | <0.0005 |
| **Localization** |  |  |  |
| UO | 0 |  |  |
| LO | 0.073 (-0.003 – 0.150) | 1.89 | 0.059 |
| LI | -0.344 (-0.445 – -0.243) | -6.68 | <0.0005 |
| UI | -0.660 (-0.737 – -0.584) | -16.98 | <0.0005 |
| Overlapping | -0.184 (-0.240 – -0.129) | -6.53 | <0.0005 |
| Central | 0.200 (0.102 – 0.299) | 4.01 | <0.0005 |
| Nipple | 0.315 (-0.058 – 0.688) | 1.65 | 0.098 |
| Axillary tail | 0.495 (0.242 – 0.749) | 3.83 | <0.0005 |
| **Age** | -0.009 (-0.011 – -0.007) | -10.49 | <0.0005 |
| **Grade** |  |  |  |
| G1 | 0 |  |  |
| G2 | 0.643 (0.570 – 0.717) | 17.15 | <0.0005 |
| G3 | 0.860 (0.782 – 0.939) | 21.50 | <0.0005 |
| **Subtype** |  |  |  |
| HR^+^/HER2^+^ | 0 |  |  |
| HR^+^/HER2^-^ | -0.094 (-0.162 – 0.025) | -2.70 | 0.007 |
| HR^-^/HER2^+^ | 0.053 (-0.060 – 0.165) | 0.92 | 0.359 |
| HR^-^/HER2^-^ | -0.598 (-0.688 – 0.508) | -13.07 | <0.0005 |

Legend: UO – upper outer quadrant; LO – lower outer quadrant; LI – lower inner quadrant; UI – upper inner quadrant.

**Supplementary Table 7.** Predictors of two or more positive lymph node by logistic regression in initial BCS patients (*n*=44,151)

| **Factor** | **Coef. (95% CI)** | **Z** | ***p*** |
| --- | --- | --- | --- |
| **Constant** | -3.236 (-3.456 – -3.015) | -28.72 | <0.0005 |
| **Tumor size** | 0.083 (0.079 – 0.086) | 46.52 | <0.0005 |
| **Localization** |  |  |  |
| UO | 0 |  |  |
| LO | 0.057 (-0.056 – 0.170) | 0.99 | 0.323 |
| LI | -0.317 (-0.463 – -0.170) | -4.24 | <0.0005 |
| UI | -0.759 (-0.873 – -0.645) | -13.03 | <0.0005 |
| Overlapping | -0.243 (-0.325 – -0.161) | -5.80 | <0.0005 |
| Central | -0.005 (-0.185 – 0.175) | -0.05 | 0.957 |
| Nipple | 0.205 (-0.457 – 0.867) | 0.61 | 0.544 |
| Axillary tail | 0.707 (0.400 – 1.013) | 4.52 | <0.0005 |
| **Age** | -0.010 (-0.013 – -0.007) | -7.19 | <0.0005 |
| **Grade** |  |  |  |
| G1 | 0 |  |  |
| G2 | 0.483 (0.384 – 0.583) | 9.45 | <0.0005 |
| G3 | 0.663 (0.553 – 0.773) | 11.81 | <0.0005 |
| **Subtype** |  |  |  |
| HR^+^/HER2^+^ | 0 |  |  |
| HR^+^/HER2^-^ | -0.094 (-0.200 – 0.012) | -1.73 | 0.084 |
| HR^-^/HER2^+^ | -0.040 (-0.232 – 0.150) | -0.42 | 0.678 |
| HR^-^/HER2^-^ | -0.579 (-0.712 – 0.439) | -8.12 | <0.0005 |

Legend: UO – upper outer quadrant; LO – lower outer quadrant; LI – lower inner quadrant; UI – upper inner quadrant.
